# A lanthanum complex with 3,4,5,6-tetra­bromophthalate as ligand

**DOI:** 10.1107/S2056989026008340

**Published:** 2026-08-28

**Authors:** Christine Hénaff, Thierry Roisnel, Chloé Blais, Olivier Guillou, Carole Daiguebonne

**Affiliations:** a Univ Rennes, INSA Rennes, CNRS UMR 6226 "Institut des Sciences Chimiques de Rennes", 35708 Rennes, France; b Univ Rennes, CNRS UMR 6226, "Institut des Sciences Chimiques de Rennes", 35042 Rennes, France; c Institut Universitaire de France, 1 rue Descartes 75005 Paris France; Universidade Federal do ABC, Brazil

**Keywords:** crystal structure, lanthanum, 3,4,5,6-tetra­bromo-phthalate, complex, luminescent marker

## Abstract

A lanthanum-based complex, heptaaqua(3,4,5,6-tetrabromobenzene-1,2-dicarboxylato)(3,4,5,6-tetrabromo-2-carboxybenzoato)lanthanum(III), [La(C_8_Br_4_O_4_)(C_8_HBr_4_O_4_)(H_2_O)_7_] or [La(tbpa)(Htbpa)(H_2_O)_7_], where H_2_tbpa is 3,4,5,6-tetra­bromo­phthalic acid, was prepared by microwave-assisted reaction between lanthanum chloride and 3,4,5,6-tetra­bromo­benzene-1,2-di­carb­oxy­lic or tetra­bromo­phthalic acid in water. The La^3+^ is nine-coordinated by seven oxygen atoms from coordinated water mol­ecules and two oxygen atoms from two different ligands in a slightly distorted *D*_3*h*_ spherical tricapped trigonal prismatic geometry. The crystal packing features a hydrogen-bonding network and Br⋯Br inter­actions.

## Abstract 2

A lanthanum-based complex with chemical formula [La(tbpa)(Htbpa)(H_2_O)_7_] where H_2_tbpa is 3,4,5,6-tetra­bromo­phthalic acid has been prepared and structurally described.

## 1. Chemical context

Tracking materials throughout their life cycle and across the supply chain is a major challenge, whether it is to combat counterfeiting or to accurately identify the nature of a mat­erial to ensure its correct recycling. Lanthanide coordination polymers have proved effective for marking materials in bulk as part of anti-counterfeiting efforts. They could also be relevant for marking plastics in bulk and so enable rigorous waste sorting. (Daiguebonne *et al.*, 2025 ▸). Indeed, regardless of the recycling method (mechanical, chemical, or biochemical), the uniformity of waste batches is a key factor (Vollmer *et al.*, 2020 ▸). While identification in the laboratory is relevant in the anti-counterfeiting field, it must be realized on sorting lines during the recycling process. This latter technological field is thus much more demanding than the former and requires highly efficient and easily identifiable markers. The quest for highly luminescent coordination compounds usable for marking plastics therefore remains an ongoing challenge. Our group has been conducting most of its research in this field for about 20 years. These works have shown that lanthanide coordination polymers based on halogeno phthalate ligands are very promising. Indeed, these non-toxic and inexpensive compounds can be prepared in high yields in water. The energies of their first excited triplet and singlet states are suitable (Latva *et al.*, 1997 ▸; Steemers *et al.*, 1995 ▸) for ensuring effective ‘antenna effects’ (Weissman *et al.*, 1942 ▸). Additionally, their adjacent carboxyl­ate functions, which allow for rigid mol­ecular motifs and prevent the presence of water mol­ecules in the first coordination sphere of lanthanide ions, are known for being beneficial to strong luminescence (Bünzli *et al.*, 2010 ▸, 2015 ▸). Finally, halogen substituents, by establishing halogen⋯halogen inter­actions, contribute to keeping the mol­ecular motifs spaced apart and avoiding π–π inter­actions.

**Figure 1:**
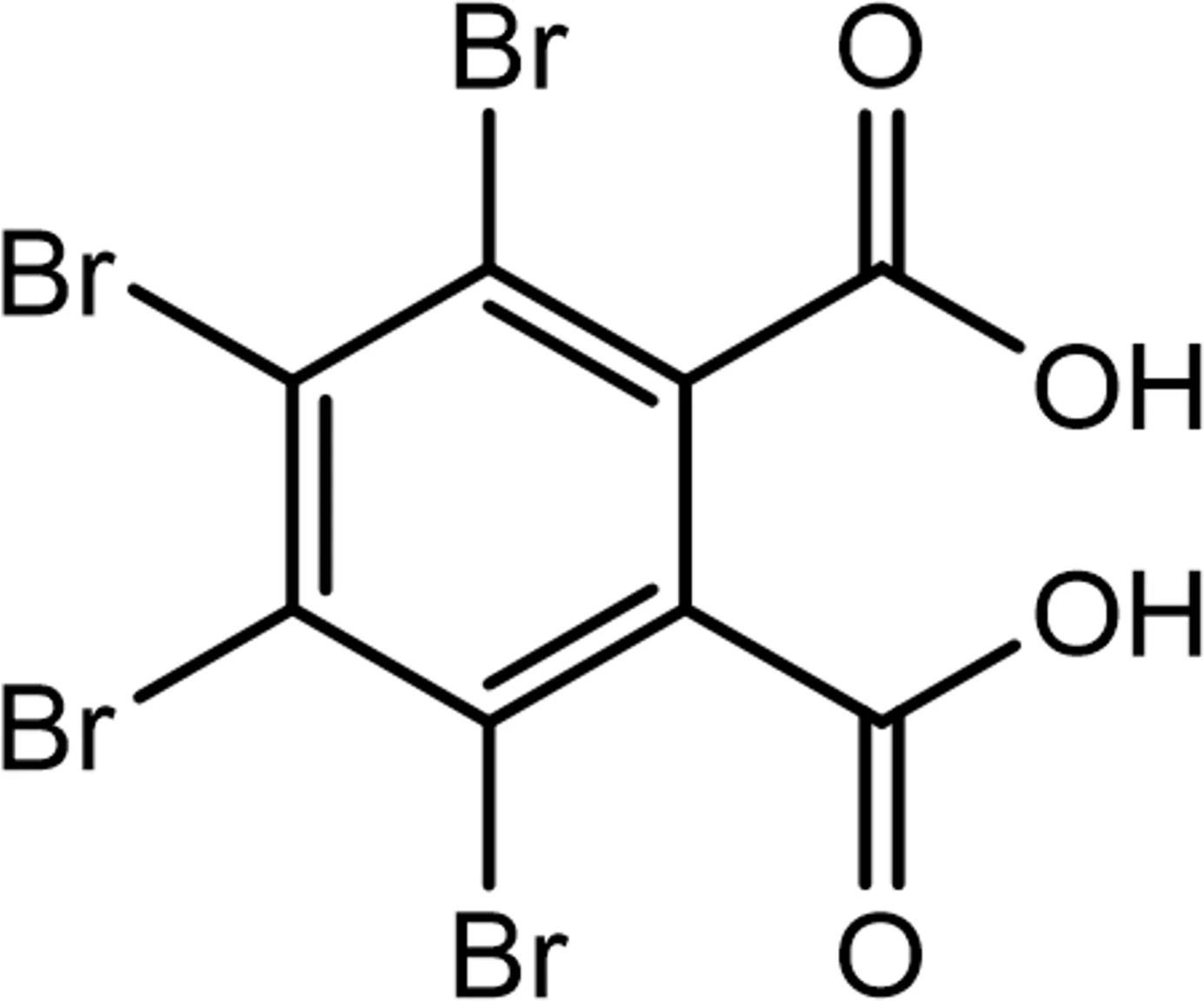
Schematic representation of 3,4,5,6-tetra­bromobenzene-1,2-di­carb­oxy­lic or tetra­bromo­phthalic acid (H_2_tbpa).

Surprisingly, studies devoted to lanthanide coordination polymers based on phthalate or halogeno phthalate are fairly rare, and those devoted to the study of their luminescent properties are even rarer (Hénaff *et al.*, 2026 ▸). To the best of our knowledge, only lanthanide coordination polymers based on di­chloro­phthalate or tetra­chloro­phthalate ligands have been studied in terms of their luminescent properties (Badiane *et al.*, 2018 ▸; Blais *et al.*, 2025 ▸, 2026 ▸; Ngom, Chang *et al.*, 2024 ▸). The studies revealed that these compounds exhibit excellent luminescence properties (Blais *et al.*, 2022 ▸). However, the number, the nature and the position on the phenyl ring of the halogen atoms have clearly a strong influence on the luminescent properties of the compounds. Indeed, they influence the energies of the first excited triplet and singlet states (Clark *et al.*, 1963 ▸), the strength of the halogen⋯halogen inter­actions (Fourmigué, 2009 ▸; Metrangolo, 2001 ▸; Cavallo *et al.*, 2016 ▸) and the steric hindrance of the ligand (Daiguebonne *et al.*, 2000 ▸). For these reasons, we undertook a systematic study of rare-earth coordination compounds based on halogeno-phthalate ligands. It was during this study that the complex [La(tbpa)(Htbpa)(H_2_O)_7_] was obtained. To the best of our knowledge, this is the first structurally characterized lanthanide coordination compound based on 3,4,5,6-tetra­bromo­phthalate (Fig. 1 ▸) as ligand.
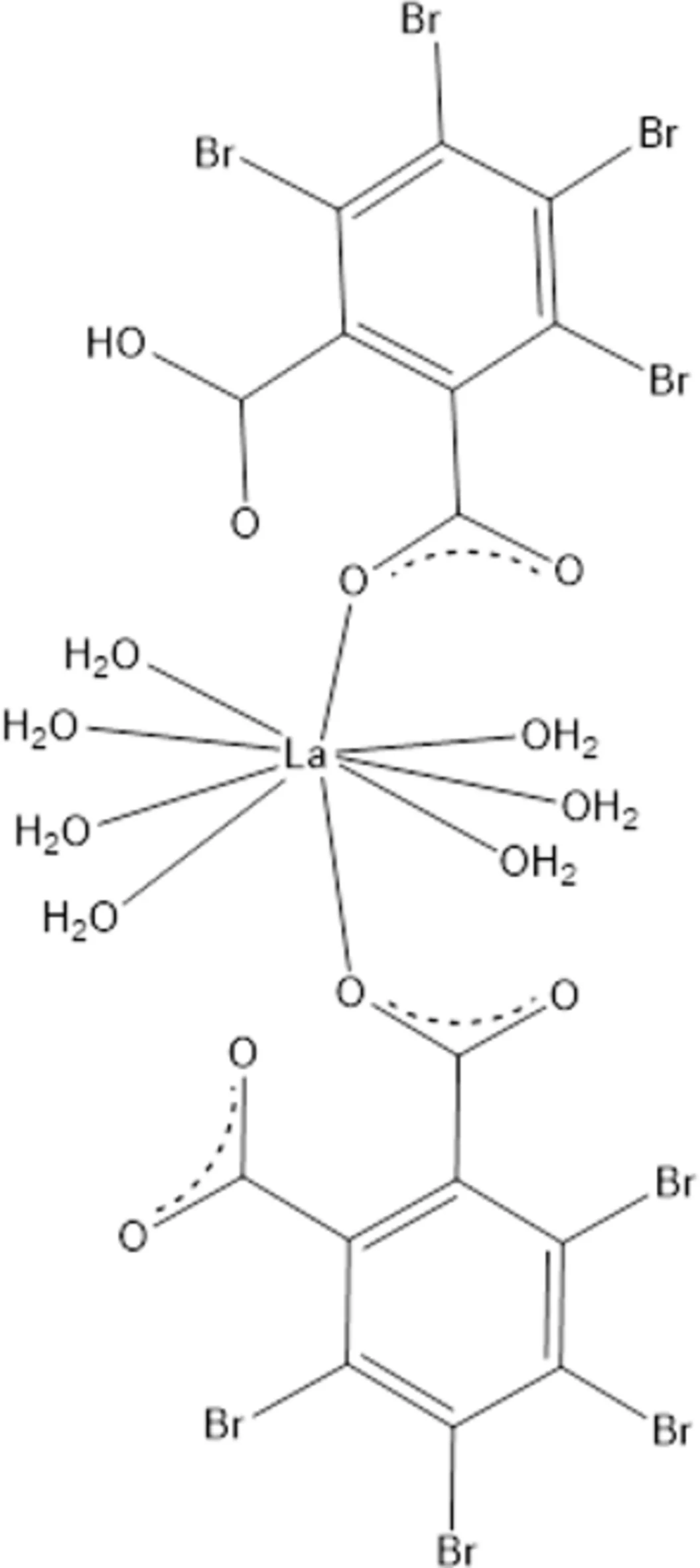


## 2. Structural commentary

**Figure 2:**
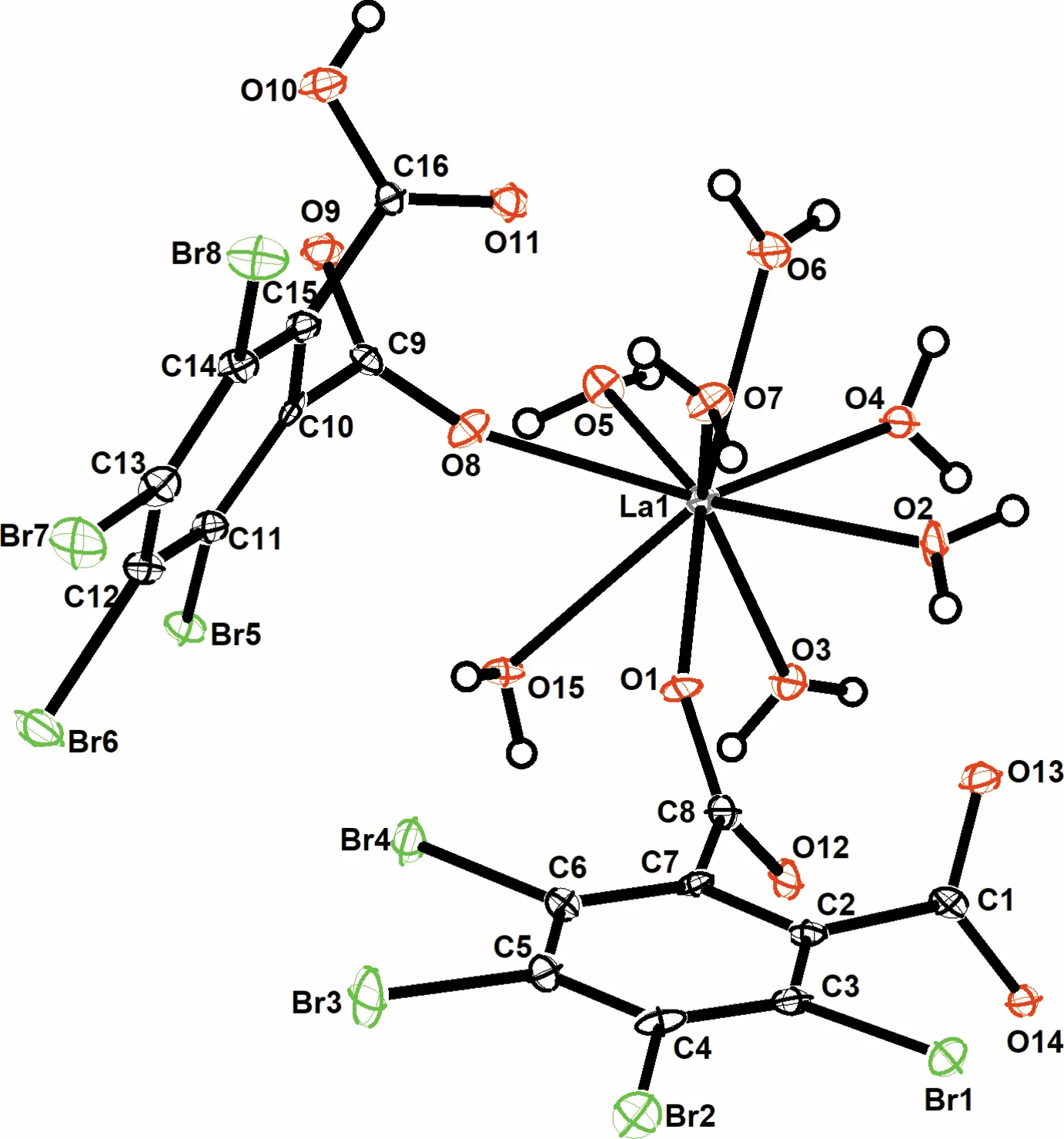
Projection view of the asymmetric unit of [La(tbpa)(Htbpa)(H_2_O)_7_] with the numbering scheme. Displacement ellipsoids are drawn at the 50% probability level.

**Figure 3:**
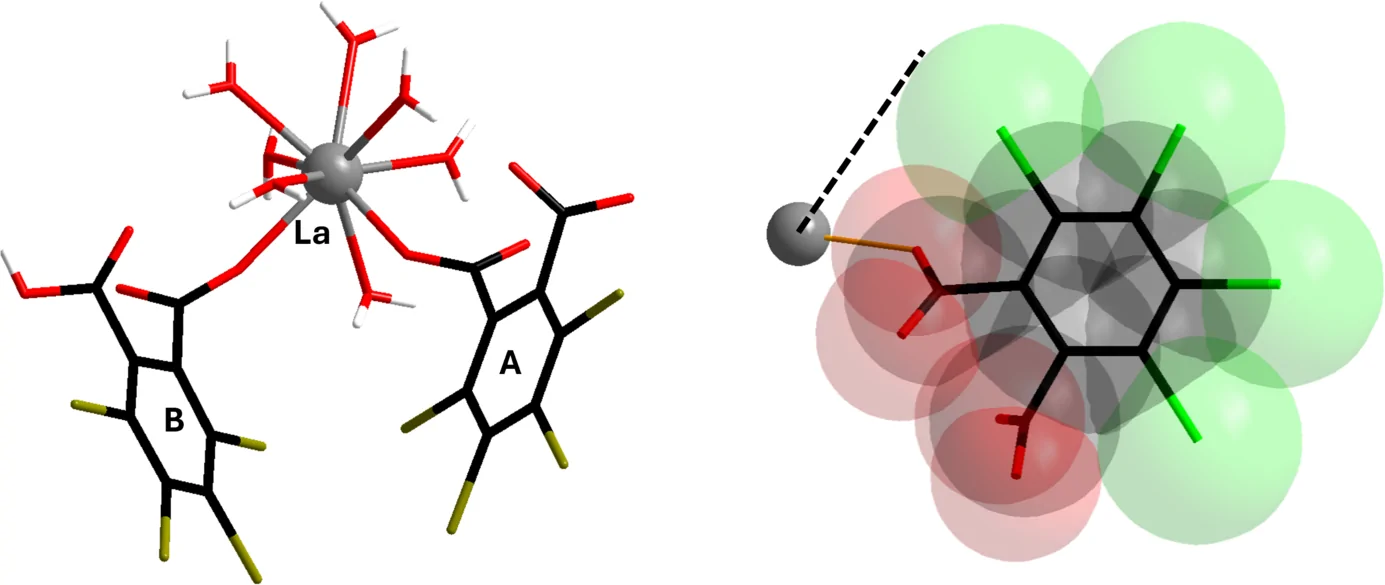
Schematic representations of the neighbourhood of the La^3+^ ion in [La(tbpa)(Htbpa)(H_2_O)_7_] (left) and of the bulky character of the tbpa^2−^ ligand (right). The dotted line highlights the steric hindrance of the ligand.

Microwave-assisted reaction in water between lanthanum chloride and 3,4,5,6-tetra­bromo-benzene-1,2-di­carb­oxy­lic or tetra­bromo­phthalic acid (hereafter denoted H_2_tbpa) leads to a lanthanum complex with chemical formula [La(tbpa)(Htbpa)(H_2_O)_7_] (Fig. 2 ▸). There is one independent La^3+^ ion in this crystal structure. It is nine-coordinated by seven oxygen atoms from coordination water mol­ecules and two oxygen atoms from two different ligands that form a slightly distorted *D*_3*h*_ spherical tricapped trigonal prism (see table in the supporting information) (Casanova *et al.*, 2005 ▸; Alvarez *et al.*, 2005 ▸). There are also two independent ligands in this crystal structure. One is fully deprotonated (type *A*) while the other one is singly protonated (type *B*). Both are μ_1_(η_1_) (Fig. 3 ▸). The high hydration rate of the lanthanide ion results from the bulky character of the ligand, which prevents it from saturating the coordination sphere of the lanthanum ion. Consequently, there are seven coordinated water mol­ecules per lanthanide ion (Daiguebonne *et al.*, 2000 ▸; Xi-Zhang *et al.*, 1987 ▸). It is noticeable that there are no water mol­ecules of crystallization in this crystal structure.

## 3. Supra­molecular features

**Figure 4:**
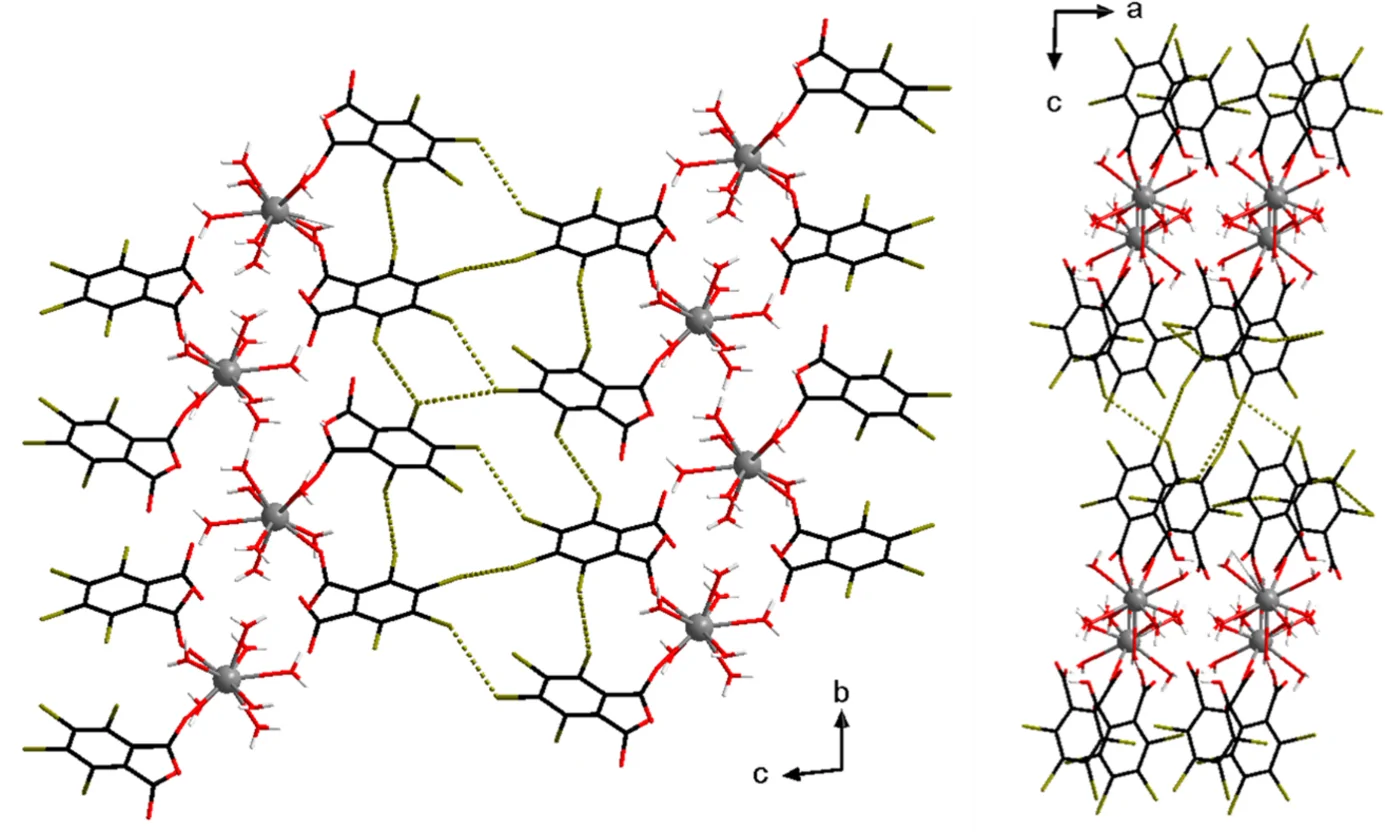
Projection views along the *a*- (left) and *b*-axis (right) of the crystal packing of [La(tbpa)(Htbpa)(H_2_O)_7_]. Dotted lines represent the shortest Br⋯Br distances.

**Figure 5:**
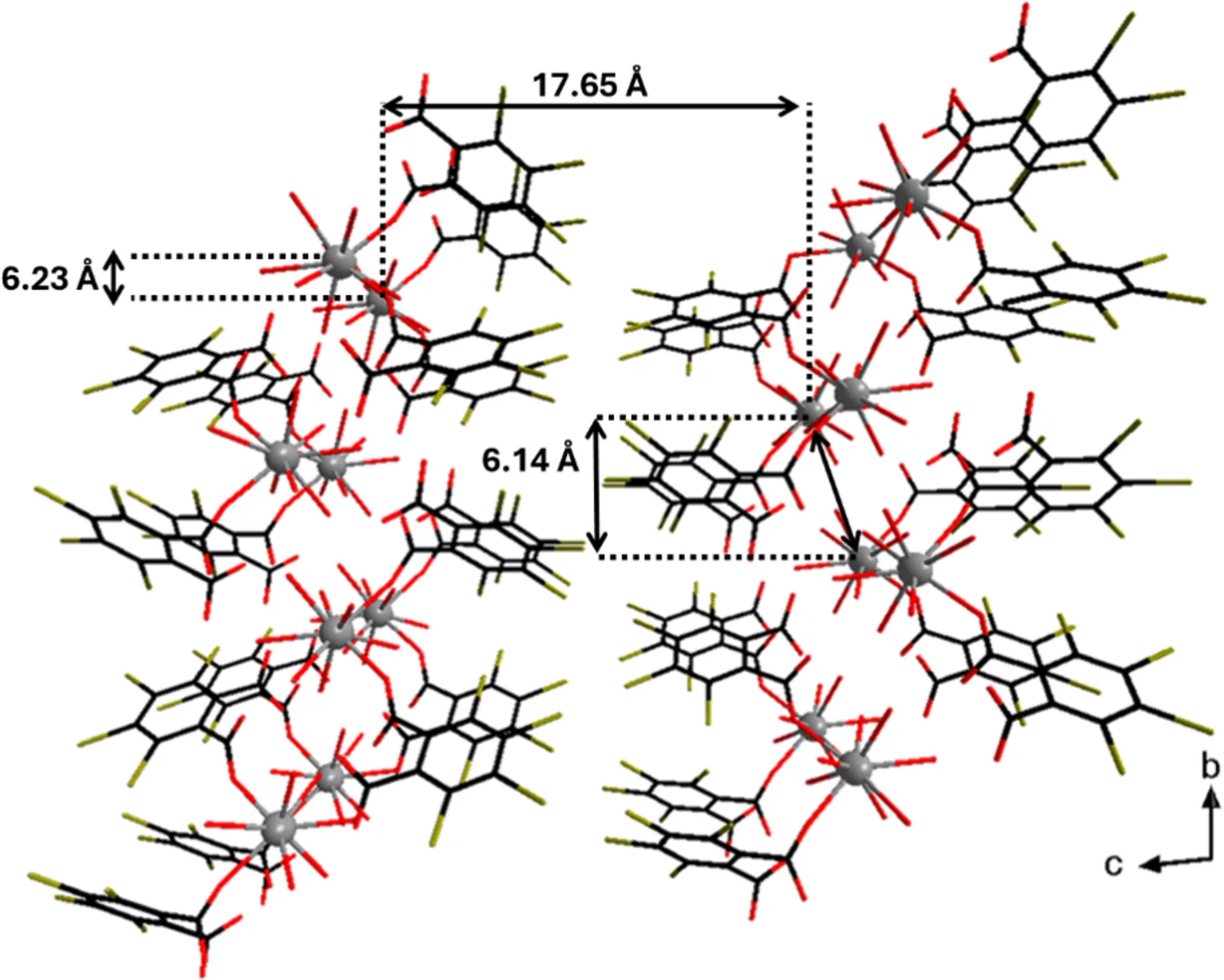
Perspective view along the *a* axis of [La(tbpa)(Htbpa)(H_2_O)_7_]. Hydrogen atoms are omitted for clarity. The shortest inter­metallic distances along the *a*, *b* and *c* axes are indicated.

The cohesion of the crystal packing is ensured by a hydrogen-bonding network (Table 1 ▸) and Br⋯Br inter­actions (Table 2 ▸). There are no π-π inter­actions in the crystal (shortest centroid–centroid distances are about 6.3 Å). As shown in Fig. 4 ▸, the bromine atoms face each other and Br⋯Br inter­actions keep the complexes far apart from one another in the *c*-axis direction. There are eight lanthanide ions closer than 10 Å, the distance above which inter­metallic energy transfers are expected to be less efficient (Imbert *et al.*, 2003 ▸) from a given lanthanide ion (Table 3 ▸ and Fig. 5 ▸). In the *c*-axis direction the shortest distances between closest lanthanide ions are all greater than 17 Å. In conclusion, the tbpa^2−^ ligand appears effective, thanks to the Br⋯Br inter­actions, at keeping the mol­ecular motifs apart from one another. This is an advantage and helps to minimize inter­metallic energy transfers. On the other hand, the steric hindrance caused by the bromine atoms is too great to allow the ligands to saturate the lanthanide ion’s coordination sphere. This results in a high number of coordinated water mol­ecules and, consequently, a high number of high-energy O—H vibrators in the vicinity of the rare earth, which is known for being detrimental to luminescence. To date, despite great synthetic efforts, we have not succeeded in preparing an isostructural compound with a luminescent rare-earth ion. It is thus not possible to experimentally evaluate the potential of this ligand for the preparation of highly luminescent compounds.

## 4. Database survey

A search of the Cambridge Structural Database was performed using ConQuest (Version 2026, CSD version 6.01, updated November 2025; Groom *et al.*, 2016 ▸). For lanthanide coordination polymers based on the tetra­chloro­phthalate ligand, see: CSD refcodes GADCOG (Chen *et al.*, 2016 ▸), TANDUK (Ma *et al.*, 2017 ▸), XOYJII (Ngom, Chang *et al.*, 2024 ▸), MUFMOT (Ngom, Blais *et al.*, 2024 ▸) and QUWSIO (Blais *et al.*, 2025 ▸). For a structural comparison between the crystal structures of phthalate and halogeno phthalate-based coordination polymers, see Hénaff *et al.* (2026 ▸).

## 5. Synthesis and crystallization

Lanthanum oxide (4 *N*) was purchased from Ampère. Hydrated lanthanum chloride [LaCl_3_(H_2_O)_6_] was prepared according to established procedures (Desreux, 1989 ▸). 3,4,5,6-Tetra­bromo­phthalic acid (H_2_tbpa) (98.75%) was purchased from BDLpharm and used without further purification. 0.4 mmol (148.5 mg) of LaCl_3_(H_2_O)_6_, 0.6 mmol (278.2 mg) of H_2_tbpa, 1.2 mL of a solution of sodium hydroxide (1 mol L^−1^) and 3.8 mL of deionized water were placed in a 10 mL sealed Pyrex test tube in a CEM Discover microwave oven and maintained for 30 min under stirring (*T* = 403 K; *P* = 2.5 bar). Single crystals suitable for X-ray diffraction analysis were obtained after slow evaporation of the supernatant solution extracted after the synthesis.

## 6. Refinement

Crystal data, data collection and structure refinement details are summarized in Table 4 ▸. The H atoms of the water mol­ecules were located and refined using a mixed model with the AFIX 2 instruction. DFIX [O—H = 0.85 (1) Å] and DANG [H⋯H = 1.37 (1) Å] restraints were applied. The hydroxyl H atom was refined using AFIX 147. The isotropic displacement parameters for all O-bound H atoms were *U*_iso_(H) = 1.2*U*_eq_(O).

## Supplementary Material

Crystal structure: contains datablock(s) global, I. DOI: 10.1107/S2056989026008340/ee2031sup1.cif

Structure factors: contains datablock(s) I. DOI: 10.1107/S2056989026008340/ee2031Isup2.hkl

CCDC reference: 2430860

Additional supporting information:  crystallographic information; 3D view; checkCIF report

## Figures and Tables

**Table 1:** Hydrogen-bond geometry (Å, °)

*D*—H⋯*A*	*D*—H	H⋯*A*	*D*⋯*A*	*D*—H⋯*A*
O2—H2*A*⋯O13	0.85 (1)	2.25 (1)	3.093 (6)	175 (6)
O2—H2*B*⋯O13^v^	0.85 (1)	2.44 (5)	3.026 (6)	126 (5)
O2—H2*B*⋯O14^v^	0.85 (1)	1.89 (1)	2.729 (6)	170 (5)
O3—H3*A*⋯O13^vi^	0.85 (1)	1.88 (2)	2.701 (6)	163 (6)
O3—H3*B*⋯O12^vii^	0.85 (1)	1.91 (2)	2.700 (6)	154 (5)
O4—H4*A*⋯O9^viii^	0.85 (1)	2.00 (2)	2.794 (5)	155 (5)
O4—H4*B*⋯O12^vii^	0.85 (1)	1.99 (2)	2.803 (5)	162 (5)
O5—H5*A*⋯O7^vi^	0.85 (1)	2.06 (2)	2.881 (6)	161 (5)
O5—H5*B*⋯O11^viii^	0.85 (1)	2.26 (4)	2.990 (6)	144 (5)
O6—H6*A*⋯O5^viii^	0.85 (1)	2.01 (2)	2.830 (6)	163 (5)
O6—H6*B*⋯O9^viii^	0.85 (1)	1.78 (1)	2.619 (6)	173 (7)
O7—H7*A*⋯O15^ix^	0.85 (1)	2.07 (3)	2.835 (6)	149 (6)
O7—H7*B*⋯O11	0.85 (1)	1.97 (2)	2.806 (6)	166 (5)
O10—H10⋯O14^x^	0.84	1.79	2.577 (6)	155
O15—H15*B*⋯O13^vi^	0.85 (1)	1.84 (2)	2.661 (5)	165 (5)

Symmetry codes: (v) 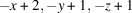; (vi) 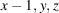; (vii) 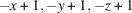; (viii) 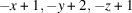; (ix) 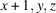; (x) 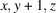.

**Table 2:** Selected interatomic distances (Å)

Br1⋯Br2	3.2865 (8)	Br3⋯Br3^iii^	3.7378 (8)
Br1⋯Br8^i^	3.6115 (8)	Br4⋯Br5	3.5336 (7)
Br2⋯Br3	3.2929 (9)	Br5⋯Br6	3.2936 (9)
Br2⋯Br7^ii^	3.5480 (9)	Br6⋯Br7	3.2977 (10)
Br2⋯Br3^ii^	3.5688 (9)	Br7⋯Br8	3.2717 (9)
Br3⋯Br4	3.2808 (8)	Br7⋯Br8^iv^	3.7295 (11)

Symmetry codes: (i) 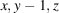; (ii) 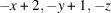; (iii) 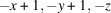; (iv) 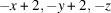.

**Table 3:** Inter­metallic distances (Å) shorter than 10Å

Atom 1	Atom 2	Symmetry	Distance
La1	La1	1 − *x*, 2 − *y*, 1 − *z*	6.1455 (4)
	La1	1 + *x*, *y*, *z*	6.2336 (4)
	La1	−1 + *x*, *y*, *z*	6.2336 (4)
	La1	1 − *x*, 1 − *y*, 1 − *z*	6.8083 (4)
	La1	2 − *x*, 2 − *y*, 1 − *z*	8.3989 (4)
	La1	−*x*, 2 − *y*, 1 − *z*	9.0945 (4)
	La1	2 − *x*, 1 − *y*, 1 − *z*	9.1469 (4)
	La1	−*x*, 1 − *y*, 1 − *z*	9.3142 (4)

**Table 4:** Experimental details

Crystal data	
Chemical formula	[La(C_8_Br_4_O_4_)(C_8_HBr_4_O_4_)(H_2_O)_7_]
*M*_r_	1225.47
Crystal system, space group	Triclinic, *P* 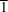
Temperature (K)	150
*a*, *b*, *c* (Å)	6.2336 (6), 12.3400 (11), 19.1394 (19)
α, β, γ (°)	96.248 (4), 90.990 (4), 91.691 (4)
*V* (Å^3^)	1462.6 (2)
*Z*	2
Radiation type	Mo *K*α
μ (mm^−1^)	12.46
Crystal size (mm)	0.54 × 0.13 × 0.07

Data collection	
Diffractometer	D8 VENTURE Bruker AXS
Absorption correction	Multi-scan (*SADABS*; Krause *et al.*, 2015 ▸)
*T*_min_, *T*_max_	0.245, 0.418
No. of measured, independent and observed [*I* > 2σ(*I*)] reflections	15668, 5917, 5164
*R*_int_	0.038
(sin θ/λ)_max_ (Å^−1^)	0.625

Refinement	
*R*[*F*^2^ > 2σ(*F*^2^)], *wR*(*F*^2^), *S*	0.035, 0.115, 1.06
No. of reflections	5917
No. of parameters	374
No. of restraints	21
H-atom treatment	H atoms treated by a mixture of independent and constrained refinement
Δρ_max_, Δρ_min_ (e Å^−3^)	1.71, −1.46

Computer programs: *APEX3* and *SAINT* (Bruker, 2015 ▸), *SHELXT* (Sheldrick, 2015*a* ▸), *SHELXL2019/2* (Sheldrick, 2015*b* ▸), *SXGRAPH* (Farrugia, 1999 ▸), *Mercury* (Macrae *et al.*, 2020 ▸) and *CRYSCALC* (T. Roisnel, local program, 2024).

## supplementary crystallographic information

### Heptaaqua(3,4,5,6-tetrabromobenzene-1,2-dicarboxylato)(3,4,5,6-tetrabromo-2-carboxybenzoato)lanthanum(III), Crystal data

**Table:**

[La(C_8_Br_4_O_4_)(C_8_HBr_4_O_4_)(H_2_O)_7_]	*Z* = 2
*M**_r_* = 1225.47	*F*(000) = 1136
Triclinic, *P*1	*D*_x_ = 2.783 Mg m^−^^3^
*a* = 6.2336 (6) Å	Mo *K*α radiation, λ = 0.71073 Å
*b* = 12.3400 (11) Å	Cell parameters from 9977 reflections
*c* = 19.1394 (19) Å	θ = 2.6–27.5°
α = 96.248 (4)°	µ = 12.46 mm^−^^1^
β = 90.990 (4)°	*T* = 150 K
γ = 91.691 (4)°	Board, colourless
*V* = 1462.6 (2) Å^3^	0.54 × 0.13 × 0.07 mm

### Heptaaqua(3,4,5,6-tetrabromobenzene-1,2-dicarboxylato)(3,4,5,6-tetrabromo-2-carboxybenzoato)lanthanum(III), Data collection

**Table:**

D8 VENTURE Bruker AXS diffractometer	5917 independent reflections
Radiation source: Incoatec microfocus sealed tube	5164 reflections with *I* > 2σ(*I*)
Multilayer monochromator	*R*_int_ = 0.038
Detector resolution: 7.39 pixels mm^-1^	θ_max_ = 26.4°, θ_min_ = 2.6°
rotation images scans	*h* = −7→7
Absorption correction: multi-scan (SADABS; Krause *et al.*, 2015)	*k* = −15→15
*T*_min_ = 0.245, *T*_max_ = 0.418	*l* = −23→23
15668 measured reflections	

### Heptaaqua(3,4,5,6-tetrabromobenzene-1,2-dicarboxylato)(3,4,5,6-tetrabromo-2-carboxybenzoato)lanthanum(III), Refinement

**Table:**

Refinement on *F*^2^	Primary atom site location: dual
Least-squares matrix: full	Hydrogen site location: mixed
*R*[*F*^2^ > 2σ(*F*^2^)] = 0.035	H atoms treated by a mixture of independent and constrained refinement
*wR*(*F*^2^) = 0.115	*w* = 1/[σ^2^(*F*_o_^2^) + (0.0767*P*)^2^] where *P* = (*F*_o_^2^ + 2*F*_c_^2^)/3
*S* = 1.06	(Δ/σ)_max_ = 0.001
5917 reflections	Δρ_max_ = 1.71 e Å^−^^3^
374 parameters	Δρ_min_ = −1.46 e Å^−^^3^
21 restraints	

### Heptaaqua(3,4,5,6-tetrabromobenzene-1,2-dicarboxylato)(3,4,5,6-tetrabromo-2-carboxybenzoato)lanthanum(III), Special details

**Table:**

Geometry. All esds (except the esd in the dihedral angle between two l.s. planes) are estimated using the full covariance matrix. The cell esds are taken into account individually in the estimation of esds in distances, angles and torsion angles; correlations between esds in cell parameters are only used when they are defined by crystal symmetry. An approximate (isotropic) treatment of cell esds is used for estimating esds involving l.s. planes.
Refinement. Crystal structure was solved by dual-space algorithm using SHELXT program (Sheldrick, 2015) and then refined with full-matrix least-squares methods based on F2 (SHELXL). All non-Hydrogen atoms were refined with anisotropic atomic displacement parameters. The final refinement on *F^2^* with 6584 unique intensities and 320 parameters converged at *ωR_F_^2^*=0.1096 (R_F_=0.0349) for 5164 observed reflections with *I*>2*σ(I)*. In the CHECKCIF procedure, no A-type meaningful alert has remained Refinement of F^2^ against ALL reflections. The weighted R-factor wR and goodness of fit S are based on F^2^, conventional R-factors R are based on F, with F set to zero for negative F^2^. The threshold expression of F^2^ > 2sigma(F^2^) is used only for calculating R-factors(gt) etc. and is not relevant to the choice of reflections for refinement. R-factors based on F^2^ are statistically about twice as large as those based on F, and R- factors based on ALL data will be even larger.

### Heptaaqua(3,4,5,6-tetrabromobenzene-1,2-dicarboxylato)(3,4,5,6-tetrabromo-2-carboxybenzoato)lanthanum(III), Fractional atomic coordinates and isotropic or equivalent isotropic displacement parameters (Å^2^)

**Table:**

	*x*	*y*	*z*	*U*_iso_*/*U*_eq_	
La1	0.52218 (5)	0.75582 (2)	0.44897 (2)	0.00807 (11)	
Br1	1.30712 (9)	0.29275 (4)	0.23795 (3)	0.01532 (15)	
Br2	1.15318 (10)	0.33774 (5)	0.07884 (3)	0.02007 (16)	
Br3	0.74303 (11)	0.49409 (5)	0.05785 (3)	0.02421 (17)	
Br4	0.46824 (9)	0.58920 (5)	0.19457 (3)	0.01814 (15)	
Br5	0.10228 (9)	0.80306 (4)	0.21605 (3)	0.01595 (15)	
Br6	0.29127 (11)	0.77604 (5)	0.05568 (4)	0.02413 (17)	
Br7	0.73350 (11)	0.91220 (5)	0.02153 (4)	0.02452 (17)	
Br8	0.97681 (10)	1.07518 (5)	0.14639 (4)	0.02193 (16)	
O1	0.6451 (6)	0.6247 (3)	0.3526 (2)	0.0142 (9)	
O2	0.8468 (6)	0.6624 (3)	0.4899 (2)	0.0143 (8)	
H2A	0.914 (8)	0.616 (4)	0.463 (2)	0.017*	
H2B	0.907 (9)	0.666 (5)	0.5306 (13)	0.017*	
O3	0.3388 (7)	0.5916 (3)	0.4919 (2)	0.0123 (8)	
H3A	0.273 (9)	0.547 (4)	0.4612 (19)	0.015*	
H3B	0.326 (10)	0.567 (4)	0.5313 (13)	0.015*	
O4	0.5210 (6)	0.7546 (3)	0.5858 (2)	0.0131 (8)	
H4A	0.572 (10)	0.804 (3)	0.617 (2)	0.016*	
H4B	0.512 (10)	0.696 (2)	0.605 (3)	0.016*	
O5	0.1924 (6)	0.8589 (3)	0.5052 (2)	0.0142 (8)	
H5A	0.078 (5)	0.853 (5)	0.480 (2)	0.017*	
H5B	0.157 (8)	0.861 (5)	0.5478 (9)	0.017*	
O6	0.6957 (7)	0.9232 (3)	0.5158 (2)	0.0153 (9)	
H6A	0.721 (11)	0.985 (2)	0.501 (3)	0.018*	
H6B	0.678 (11)	0.934 (4)	0.5597 (8)	0.018*	
O7	0.8708 (6)	0.8335 (3)	0.3942 (2)	0.0150 (9)	
H7A	0.950 (9)	0.790 (3)	0.370 (3)	0.018*	
H7B	0.864 (10)	0.891 (3)	0.373 (3)	0.018*	
O8	0.4134 (6)	0.8735 (3)	0.3598 (2)	0.0159 (9)	
O9	0.3261 (6)	1.0449 (3)	0.3470 (2)	0.0151 (9)	
O10	0.7734 (7)	1.1778 (3)	0.2832 (2)	0.0168 (9)	
H10	0.813852	1.215681	0.320490	0.025*	
O11	0.8232 (6)	1.0385 (3)	0.3462 (2)	0.0147 (9)	
O12	0.5791 (6)	0.4533 (3)	0.3754 (2)	0.0139 (8)	
O13	1.0695 (6)	0.4820 (3)	0.3952 (2)	0.0155 (9)	
O14	0.9943 (6)	0.3045 (3)	0.3754 (2)	0.0126 (8)	
O15	0.1948 (6)	0.6794 (3)	0.3651 (2)	0.0130 (8)	
H15A	0.240 (10)	0.686 (4)	0.3243 (14)	0.016*	
H15B	0.157 (10)	0.6131 (16)	0.367 (3)	0.016*	
C1	1.0096 (8)	0.3987 (4)	0.3559 (3)	0.0092 (11)	
C2	0.9454 (9)	0.4158 (4)	0.2816 (3)	0.0092 (11)	
C3	1.0610 (9)	0.3744 (4)	0.2242 (3)	0.0113 (11)	
C4	1.0005 (9)	0.3963 (4)	0.1576 (3)	0.0144 (12)	
C5	0.8214 (9)	0.4612 (4)	0.1480 (3)	0.0129 (11)	
C6	0.7098 (9)	0.5039 (4)	0.2058 (3)	0.0126 (11)	
C7	0.7696 (9)	0.4808 (4)	0.2736 (3)	0.0101 (11)	
C8	0.6519 (8)	0.5225 (4)	0.3394 (3)	0.0121 (12)	
C9	0.3958 (8)	0.9535 (4)	0.3245 (3)	0.0106 (11)	
C10	0.4690 (9)	0.9412 (4)	0.2493 (3)	0.0125 (5)	
C11	0.3619 (9)	0.8763 (4)	0.1949 (3)	0.0125 (5)	
C12	0.4355 (9)	0.8674 (4)	0.1269 (3)	0.0125 (5)	
C13	0.6226 (9)	0.9271 (4)	0.1119 (3)	0.0125 (5)	
C14	0.7255 (9)	0.9954 (4)	0.1665 (3)	0.0125 (5)	
C15	0.6521 (9)	1.0039 (4)	0.2334 (3)	0.0125 (5)	
C16	0.7609 (9)	1.0747 (4)	0.2941 (3)	0.0109 (11)	

### Heptaaqua(3,4,5,6-tetrabromobenzene-1,2-dicarboxylato)(3,4,5,6-tetrabromo-2-carboxybenzoato)lanthanum(III), Atomic displacement parameters (Å^2^)

**Table:**

	*U*^11^	*U*^22^	*U*^33^	*U*^12^	*U*^13^	*U*^23^
La1	0.00835 (17)	0.00649 (15)	0.00928 (19)	0.00016 (11)	0.00035 (12)	0.00048 (12)
Br1	0.0131 (3)	0.0155 (3)	0.0173 (3)	0.0043 (2)	0.0011 (2)	0.0000 (2)
Br2	0.0261 (3)	0.0211 (3)	0.0133 (3)	0.0064 (2)	0.0075 (3)	0.0005 (2)
Br3	0.0308 (4)	0.0331 (3)	0.0101 (3)	0.0107 (3)	0.0012 (3)	0.0059 (3)
Br4	0.0195 (3)	0.0194 (3)	0.0163 (3)	0.0087 (2)	−0.0011 (2)	0.0034 (2)
Br5	0.0141 (3)	0.0150 (3)	0.0184 (3)	−0.0033 (2)	−0.0028 (2)	0.0019 (2)
Br6	0.0313 (4)	0.0246 (3)	0.0142 (4)	−0.0064 (3)	−0.0047 (3)	−0.0048 (3)
Br7	0.0298 (4)	0.0292 (3)	0.0137 (4)	0.0022 (3)	0.0072 (3)	−0.0026 (3)
Br8	0.0239 (3)	0.0205 (3)	0.0208 (4)	−0.0069 (2)	0.0097 (3)	−0.0003 (3)
O1	0.016 (2)	0.0081 (17)	0.017 (2)	0.0038 (14)	0.0007 (17)	−0.0057 (16)
O2	0.017 (2)	0.022 (2)	0.005 (2)	0.0062 (16)	−0.0017 (17)	0.0027 (17)
O3	0.020 (2)	0.0102 (18)	0.007 (2)	−0.0027 (15)	0.0005 (17)	0.0023 (15)
O4	0.019 (2)	0.0090 (17)	0.010 (2)	−0.0020 (15)	−0.0033 (17)	0.0009 (16)
O5	0.0106 (19)	0.0183 (19)	0.013 (2)	−0.0006 (15)	0.0032 (17)	−0.0013 (17)
O6	0.026 (2)	0.0124 (18)	0.008 (2)	−0.0021 (16)	0.0027 (19)	0.0013 (16)
O7	0.016 (2)	0.0130 (18)	0.016 (2)	0.0015 (15)	0.0042 (18)	0.0014 (17)
O8	0.016 (2)	0.0117 (18)	0.021 (3)	0.0002 (15)	0.0028 (18)	0.0059 (17)
O9	0.019 (2)	0.0115 (18)	0.014 (2)	0.0028 (15)	0.0015 (18)	−0.0006 (16)
O10	0.022 (2)	0.0092 (18)	0.018 (3)	−0.0025 (15)	0.0006 (19)	−0.0005 (17)
O11	0.017 (2)	0.0141 (19)	0.012 (2)	−0.0035 (15)	−0.0027 (17)	0.0025 (17)
O12	0.018 (2)	0.0140 (18)	0.010 (2)	−0.0020 (15)	−0.0005 (17)	0.0030 (16)
O13	0.017 (2)	0.0088 (18)	0.020 (3)	−0.0020 (15)	−0.0025 (18)	0.0017 (16)
O14	0.017 (2)	0.0088 (17)	0.012 (2)	−0.0020 (14)	−0.0022 (17)	0.0021 (15)
O15	0.015 (2)	0.0085 (17)	0.014 (2)	−0.0040 (14)	−0.0011 (17)	−0.0018 (16)
C1	0.006 (2)	0.015 (3)	0.006 (3)	0.0026 (19)	0.002 (2)	−0.003 (2)
C2	0.015 (3)	0.007 (2)	0.005 (3)	−0.0017 (19)	0.001 (2)	−0.002 (2)
C3	0.013 (3)	0.008 (2)	0.013 (3)	0.0002 (19)	0.001 (2)	−0.003 (2)
C4	0.015 (3)	0.008 (2)	0.019 (3)	0.000 (2)	0.007 (2)	−0.003 (2)
C5	0.021 (3)	0.015 (3)	0.004 (3)	−0.003 (2)	0.000 (2)	0.003 (2)
C6	0.014 (3)	0.011 (2)	0.012 (3)	−0.001 (2)	−0.003 (2)	−0.002 (2)
C7	0.013 (3)	0.005 (2)	0.012 (3)	−0.0003 (18)	0.004 (2)	0.002 (2)
C8	0.008 (2)	0.010 (2)	0.018 (3)	0.0012 (19)	−0.003 (2)	0.000 (2)
C9	0.010 (3)	0.010 (2)	0.011 (3)	−0.0023 (19)	−0.004 (2)	−0.001 (2)
C10	0.0159 (11)	0.0098 (10)	0.0117 (14)	0.0017 (9)	0.0009 (9)	0.0012 (9)
C11	0.0159 (11)	0.0098 (10)	0.0117 (14)	0.0017 (9)	0.0009 (9)	0.0012 (9)
C12	0.0159 (11)	0.0098 (10)	0.0117 (14)	0.0017 (9)	0.0009 (9)	0.0012 (9)
C13	0.0159 (11)	0.0098 (10)	0.0117 (14)	0.0017 (9)	0.0009 (9)	0.0012 (9)
C14	0.0159 (11)	0.0098 (10)	0.0117 (14)	0.0017 (9)	0.0009 (9)	0.0012 (9)
C15	0.0159 (11)	0.0098 (10)	0.0117 (14)	0.0017 (9)	0.0009 (9)	0.0012 (9)
C16	0.014 (3)	0.013 (2)	0.005 (3)	−0.001 (2)	0.007 (2)	−0.001 (2)

### Heptaaqua(3,4,5,6-tetrabromobenzene-1,2-dicarboxylato)(3,4,5,6-tetrabromo-2-carboxybenzoato)lanthanum(III), Geometric parameters (Å, º)

**Table:**

La1—O8	2.457 (4)	O7—H7A	0.851 (10)
La1—O1	2.466 (4)	O7—H7B	0.853 (10)
La1—O2	2.513 (4)	O8—C9	1.262 (7)
La1—O6	2.514 (4)	O9—C9	1.256 (7)
La1—O3	2.519 (4)	O10—C16	1.312 (6)
La1—O4	2.621 (4)	O10—H10	0.8400
La1—O7	2.629 (4)	O11—C16	1.199 (7)
La1—O5	2.633 (4)	O12—C8	1.236 (7)
La1—O15	2.660 (4)	O13—C1	1.249 (7)
Br1—C3	1.890 (5)	O14—C1	1.261 (7)
Br2—C4	1.886 (6)	O15—H15A	0.846 (10)
Br3—C5	1.876 (6)	O15—H15B	0.847 (10)
Br4—C6	1.885 (6)	C1—C2	1.509 (8)
Br5—C11	1.901 (6)	C2—C3	1.384 (8)
Br6—C12	1.871 (6)	C2—C7	1.393 (7)
Br7—C13	1.866 (6)	C3—C4	1.379 (9)
Br8—C14	1.893 (6)	C4—C5	1.414 (8)
O1—C8	1.261 (6)	C5—C6	1.380 (9)
O2—H2A	0.850 (10)	C6—C7	1.404 (8)
O2—H2B	0.853 (10)	C7—C8	1.518 (8)
O3—H3A	0.849 (10)	C9—C10	1.510 (8)
O3—H3B	0.848 (10)	C10—C11	1.392 (8)
O4—H4A	0.851 (10)	C10—C15	1.416 (8)
O4—H4B	0.848 (10)	C11—C12	1.382 (9)
O5—H5A	0.851 (10)	C12—C13	1.411 (8)
O5—H5B	0.848 (10)	C13—C14	1.403 (8)
O6—H6A	0.849 (10)	C14—C15	1.361 (9)
O6—H6B	0.847 (10)	C15—C16	1.513 (8)
			
Br1···Br2	3.2865 (8)	Br3···Br3^iii^	3.7378 (8)
Br1···Br8^i^	3.6115 (8)	Br4···Br5	3.5336 (7)
Br2···Br3	3.2929 (9)	Br5···Br6	3.2936 (9)
Br2···Br7^ii^	3.5480 (9)	Br6···Br7	3.2977 (10)
Br2···Br3^ii^	3.5688 (9)	Br7···Br8	3.2717 (9)
Br3···Br4	3.2808 (8)	Br7···Br8^iv^	3.7295 (11)
			
O8—La1—O1	88.06 (14)	H7A—O7—H7B	106.7 (17)
O8—La1—O2	140.46 (13)	C9—O8—La1	163.0 (4)
O1—La1—O2	70.66 (14)	C16—O10—H10	109.5
O8—La1—O6	87.13 (14)	La1—O15—H15A	104 (4)
O1—La1—O6	132.78 (13)	La1—O15—H15B	116 (4)
O2—La1—O6	84.11 (14)	H15A—O15—H15B	107.9 (17)
O8—La1—O3	131.38 (14)	O13—C1—O14	124.1 (5)
O1—La1—O3	84.75 (13)	O13—C1—C2	116.3 (5)
O2—La1—O3	80.80 (14)	O14—C1—C2	119.6 (5)
O6—La1—O3	130.72 (14)	C3—C2—C7	121.1 (5)
O8—La1—O4	139.16 (13)	C3—C2—C1	122.2 (5)
O1—La1—O4	132.78 (13)	C7—C2—C1	116.7 (5)
O2—La1—O4	69.60 (13)	C4—C3—C2	119.8 (5)
O6—La1—O4	66.05 (13)	C4—C3—Br1	120.5 (4)
O3—La1—O4	64.70 (13)	C2—C3—Br1	119.7 (5)
O8—La1—O7	71.72 (13)	C3—C4—C5	120.2 (5)
O1—La1—O7	69.98 (13)	C3—C4—Br2	120.2 (4)
O2—La1—O7	69.80 (13)	C5—C4—Br2	119.7 (5)
O6—La1—O7	63.94 (13)	C6—C5—C4	119.5 (6)
O3—La1—O7	146.03 (13)	C6—C5—Br3	120.2 (5)
O4—La1—O7	117.16 (13)	C4—C5—Br3	120.2 (4)
O8—La1—O5	76.26 (14)	C5—C6—C7	120.4 (5)
O1—La1—O5	145.94 (13)	C5—C6—Br4	120.5 (5)
O2—La1—O5	137.90 (14)	C7—C6—Br4	119.0 (4)
O6—La1—O5	77.17 (13)	C2—C7—C6	119.0 (5)
O3—La1—O5	83.49 (13)	C2—C7—C8	117.5 (5)
O4—La1—O5	68.36 (13)	C6—C7—C8	123.5 (5)
O7—La1—O5	129.93 (12)	O12—C8—O1	126.9 (6)
O8—La1—O15	64.43 (13)	O12—C8—C7	116.9 (5)
O1—La1—O15	69.18 (13)	O1—C8—C7	116.1 (5)
O2—La1—O15	130.61 (13)	O9—C9—O8	125.4 (6)
O6—La1—O15	145.15 (13)	O9—C9—C10	116.2 (5)
O3—La1—O15	68.11 (13)	O8—C9—C10	118.4 (5)
O4—La1—O15	123.20 (13)	C11—C10—C15	118.6 (6)
O7—La1—O15	119.62 (13)	C11—C10—C9	123.9 (5)
O5—La1—O15	76.78 (13)	C15—C10—C9	117.4 (5)
C8—O1—La1	137.1 (4)	C12—C11—C10	121.8 (5)
La1—O2—H2A	123 (4)	C12—C11—Br5	120.3 (4)
La1—O2—H2B	130 (4)	C10—C11—Br5	117.8 (5)
H2A—O2—H2B	107.0 (17)	C11—C12—C13	119.2 (5)
La1—O3—H3A	117 (3)	C11—C12—Br6	120.5 (4)
La1—O3—H3B	136 (3)	C13—C12—Br6	120.3 (5)
H3A—O3—H3B	107.7 (17)	C14—C13—C12	118.7 (6)
La1—O4—H4A	127 (4)	C14—C13—Br7	121.0 (4)
La1—O4—H4B	122 (4)	C12—C13—Br7	120.3 (4)
H4A—O4—H4B	107.4 (17)	C15—C14—C13	121.8 (5)
La1—O5—H5A	115 (4)	C15—C14—Br8	119.5 (4)
La1—O5—H5B	125 (4)	C13—C14—Br8	118.7 (4)
H5A—O5—H5B	107.5 (17)	C14—C15—C10	119.8 (5)
La1—O6—H6A	127 (4)	C14—C15—C16	123.5 (5)
La1—O6—H6B	119 (4)	C10—C15—C16	116.7 (5)
H6A—O6—H6B	107.8 (17)	O11—C16—O10	125.2 (5)
La1—O7—H7A	119 (4)	O11—C16—C15	122.4 (5)
La1—O7—H7B	119 (4)	O10—C16—C15	112.4 (5)

Symmetry codes: (i) *x*, *y*−1, *z*; (ii) −*x*+2, −*y*+1, −*z*; (iii) −*x*+1, −*y*+1, −*z*; (iv) −*x*+2, −*y*+2, −*z*.

### Heptaaqua(3,4,5,6-tetrabromobenzene-1,2-dicarboxylato)(3,4,5,6-tetrabromo-2-carboxybenzoato)lanthanum(III), Hydrogen-bond geometry (Å, º)

**Table:**

*D*—H···*A*	*D*—H	H···*A*	*D*···*A*	*D*—H···*A*
O2—H2*A*···O13	0.85 (1)	2.25 (1)	3.093 (6)	175 (6)
O2—H2*B*···O13^v^	0.85 (1)	2.44 (5)	3.026 (6)	126 (5)
O2—H2*B*···O14^v^	0.85 (1)	1.89 (1)	2.729 (6)	170 (5)
O3—H3*A*···O13^vi^	0.85 (1)	1.88 (2)	2.701 (6)	163 (6)
O3—H3*B*···O12^vii^	0.85 (1)	1.91 (2)	2.700 (6)	154 (5)
O4—H4*A*···O9^viii^	0.85 (1)	2.00 (2)	2.794 (5)	155 (5)
O4—H4*B*···O12^vii^	0.85 (1)	1.99 (2)	2.803 (5)	162 (5)
O5—H5*A*···O7^vi^	0.85 (1)	2.06 (2)	2.881 (6)	161 (5)
O5—H5*B*···O11^viii^	0.85 (1)	2.26 (4)	2.990 (6)	144 (5)
O6—H6*A*···O5^viii^	0.85 (1)	2.01 (2)	2.830 (6)	163 (5)
O6—H6*B*···O9^viii^	0.85 (1)	1.78 (1)	2.619 (6)	173 (7)
O7—H7*A*···O15^ix^	0.85 (1)	2.07 (3)	2.835 (6)	149 (6)
O7—H7*B*···O11	0.85 (1)	1.97 (2)	2.806 (6)	166 (5)
O10—H10···O14^x^	0.84	1.79	2.577 (6)	155
O15—H15*B*···O13^vi^	0.85 (1)	1.84 (2)	2.661 (5)	165 (5)

Symmetry codes: (v) −*x*+2, −*y*+1, −*z*+1; (vi) *x*−1, *y*, *z*; (vii) −*x*+1, −*y*+1, −*z*+1; (viii) −*x*+1, −*y*+2, −*z*+1; (ix) *x*+1, *y*, *z*; (x) *x*, *y*+1, *z*.

### Continuous Shape Measurements (CShM) for La(tbpa)(Htbpa)(H_2_O)_7_. The lower is the CShM value, the better is the agreement with the given coordination polyhedron

**Table:**

[ML9]	EP-9	OPY-9	HBPY-9	JTC-9	JCCU-9	CCU-9	JCSAPR-9	TCTPR-9	JTDIC-9	HH-9	MFF-9
La	33.003	24.175	18.551	14.980	8.289	7.091	1.768	1.262	13.342	9.394	1.742

EP-9 ≡*D*_9_*_h_*-Enneagon; OPY-9 ≡*C*_8_*_v_*-Octagonal pyramid; HBPY-9 ≡*D*_7_*_h_*-Heptagonal bipyramid; JTC-9 ≡*C*_3_*_v_*-Johnson triangular cupola J3; JCCU-9 ≡*C*_4_*_v_*-Capped cube J8; CCU-9 ≡*C*_4_*_v_*-Spherical-relaxed capped cube; JCSAPR-9 ≡*C*_4_*_v_*-Capped square antiprism J10; CSAPR-9 ≡*C*_4_*_v_*-Spherical capped square antiprism; JTCTPR-9 ≡*D*_3_*_h_*-Tricapped trigonal prism J51; TCTPR-9 ≡*D*_3_*_h_*-Spherical tricapped trigonal prism; JTDIC-9 ≡*C*_3_*_v_*-Tridiminished icosahedron J63; HH-9 ≡*C*_2_*_v_*-Hula-hoop; MFF-9 ≡*C_s_*-Muffin.
